# Sensor-integrated industrial exoskeletons for ergonomic risk reduction: a systematic review of biomechanical outcomes and intelligent control systems

**DOI:** 10.3389/fbioe.2026.1952925

**Published:** 2026-10-22

**Authors:** Rusber Alberto Risco-Ojeda, Cesar Moreno-Rojo, Ruben Adrián Figueroa-León, Saúl Ricardo Chuqui-Diestra, Juan Carlos Ponce-Ramirez, Edgar Virgilio Bedoya-Justo, Nelver Javier Escalante-Espinoza, Wendy Akemmy Castañeda-Rodríguez, Abel José Rodríguez-Yparraguirre, Carlos Diego Rodríguez-Yparraguirre

**Affiliations:** 1 Department of Energy, Physics and Mechanics, Faculty of Engineering, Universidad Nacional del Santa, Chimbote, Ancash, Peru; 2 Department of Agroindustry and Agronomy, Faculty of Engineering, Universidad Nacional del Santa, Chimbote, Ancash, Peru; 3 Department of Chemical Engineering, Faculty of Chemical and Metallurgical Engineering, Universidad Nacional de San Cristóbal de Huamanga, Ayacucho, Peru; 4 Department of Environmental Engineering, Faculty of Engineering and Architecture, Universidad Nacional de Moquegua, Moquegua, Peru; 5 Doctoral Program in Agro-Industrial Engineering, Specialization in Advanced Processing of Andean Grains and Tubers, Universidad Nacional del Santa, Chimbote, Ancash, Peru; 6 Doctoral Program in Science and Engineering, Graduate School, Universidad Nacional de Trujillo, Trujillo, La Libertad, Peru

**Keywords:** ergonomic assessment, human-robot interaction, industry 5.0, sensor fusion, wearable sensors, work-related musculoskeletal disorders (WMSDs)

## Abstract

**Introduction:**

Industrial exoskeletons with integrated sensors are emerging as a technology for monitoring biomechanical exposure and supporting the prevention of work-related musculoskeletal disorders in industrial environments.

**Methods:**

This systematic review followed the PRISMA 2020 guidelines, retrieving 106 studies published between 2021 and 2026 from the Scopus and Web of Science databases. VOSviewer version 1.6.20 and R‐Studio version 2026.01.2+418 were used for bibliometric and descriptive analyses. The review provides a qualitative synthesis supported by descriptive quantitative reporting of the extracted outcomes.

**Results:**

The evidence indicated a technological shift from passive devices toward intelligent exoskeletons integrating surface electromyography, inertial measurement units, machine vision, physiological sensors, and artificial intelligence. Individual studies reported maximum reductions of 68.2% in muscle activity, 34% in spinal compression, and up to 60% in muscle fatigue, alongside productivity improvements of up to 59.5%. Machine learning approaches achieved an ROC of 0.985, automated assessment accuracies of 98.5%, and posture estimation with an ICC of 0.967. However, these values were not pooled estimates and varied across study designs, sensor configurations, tasks, and assessment protocols.

**Discussion:**

The evidence remains constrained by small sample sizes, sensor drift, interoperability limitations, and insufficient long‐term validation in real‐world industrial environments. Sensor‐integrated exoskeletons show potential for ergonomic monitoring and risk reduction, but their effectiveness and industrial generalizability require further validation through standardized, interoperable, and longitudinal field studies.

## 1 Introduction

Work-related musculoskeletal disorders (WMSDs) remain one of the leading occupational health challenges worldwide, representing a major cause of disability, reduced productivity, and economic losses across industrial sectors. Physically demanding activities, including manual material handling, repetitive movements, awkward postures, and overhead tasks, continue to expose workers to excessive biomechanical loads that substantially increase the risk of musculoskeletal injuries. This problem is particularly critical in industries such as manufacturing, construction, logistics, and automotive assembly, where approximately 46% of musculoskeletal disorders affect construction workers and other occupations characterized by high physical demands (Al-Khiami and Lindhard, 2026a; Naik et al., 2026; Tröster et al., 2026). Consequently, improving occupational safety while maintaining productivity has become a strategic priority, driving the adoption of innovative ergonomic interventions capable of reducing biomechanical stress without compromising operational performance (Bonakdar et al., 2025; Piovesan and Ji, 2026; Vora et al., 2026).

Among the emerging ergonomic interventions, industrial exoskeletons have gained considerable attention as wearable assistive technologies designed to reduce biomechanical loading, alleviate muscle fatigue, and support the prevention of work-related musculoskeletal disorders during physically demanding tasks. Conventional exoskeletons primarily provide passive or powered mechanical assistance through structural components, springs, actuators, or load-transfer mechanisms that redistribute physical demands across the body. In contrast, sensor-integrated exoskeletons extend this mechanical function by incorporating wearable sensing and computational technologies, such as surface electromyography, inertial measurement units, motion capture, physiological sensors, and biomechanical modeling (Rakpongsiri et al., 2025; Zhang et al., 2026). These technologies enable the objective measurement of worker–device interaction, physical workload, movement patterns, and physiological responses, thereby supporting data-informed assistance, ergonomic assessment, and, in some systems, adaptive control. This transition from mechanical support alone toward sensing-enabled and analytically informed assistance represents a distinctive technological development in industrial exoskeleton research (Gennarelli et al., 2025).

The emergence of Industry 5.0 has further has further encouraged the development of human-centered industrial technologies that combine intelligent sensing, computational biomechanics, and worker-oriented design principles (Brandt et al., 2025; Michaud et al., 2025; Muslim et al., 2025; Sado et al., 2025). In sensor-integrated exoskeleton research, this evolution is reflected in the combination of inertial measurement units, surface electromyography, computer vision, markerless motion capture, digital human modeling, biomechanical simulations, and machine learning algorithms. Rather than functioning solely as assistive devices, these systems can be understood as interconnected human-machine platforms in which sensing, data processing, and biomechanical interpretation support the assessment of physical demands and the evaluation of assistance strategies (Vox et al., 2021; Brunner et al., 2022; Ko et al., 2024). However, the integration of multiple sensing modalities does not inherently guarantee improved ergonomic outcomes, as the reliability, interpretability, and practical value of the resulting information depend on sensor configuration, validation procedures, task conditions, and the interaction between the worker and the device (Aqueveque et al., 2023).

Although sensor integration has expanded the capabilities of industrial exoskeletons, the available evidence remains fragmented across technological configurations, sensing modalities, biomechanical outcomes, and validation approaches. Previous studies have reported the use of individual or multimodal sensors to assess muscle activity, spinal loading, posture, fatigue, and worker–device interaction; however, these findings are difficult to compare because of differences in experimental protocols, task characteristics, participant populations, outcome definitions, and performance metrics (Hubaut et al., 2022; Aqueveque et al., 2023). Consequently, an integrated understanding of how sensing architectures are configured, which ergonomic outcomes are most consistently supported, how measurement and prediction models are validated, and what conditions influence their transferability to industrial practice remains insufficiently developed. This gap limits the formulation of evidence-based conclusions regarding the technological maturity, ergonomic value, and implementation requirements of sensor-integrated exoskeleton systems.

A further limitation concerns the conditions under which sensor-integrated exoskeletons have been evaluated. Many investigations have been conducted in controlled laboratory environments using standardized tasks and relatively small participant groups, which may not adequately represent the variability, duration, and operational constraints of real production setting (Chen et al., 2026; Le and Lin, 2026). This gap is particularly relevant considering that approximately 77% of workers in the United States experience work-related musculoskeletal disorders associated with repetitive physical activities (Altheimer and Schneider, 2026; Zamorano Núñez et al., 2026), while nearly 40 million workers in the European Union continue to be affected by occupational illnesses despite ongoing improvements in workplace safety standards (Alenjareghi et al., 2026; David and Volling, 2026). En industrial environments, factors such as task variability, prolonged use, environmental conditions, worker anthropometry, comfort, sensor placement, calibration stability, and compatibility with existing workflows may influence both system performance and user acceptance.

Despite the growing body of evidence, considerable variability persists regarding the effectiveness and implementation of industrial exoskeletons across manufacturing, construction, logistics, healthcare, and other physically demanding sectors (Bhat et al., 2025; Ciccarelli et al., 2025; Purushothaman and Gedara, 2025; Rahman et al., 2025; Agostinelli et al., 2026). Differences in exoskeleton designs, sensing architectures, biomechanical evaluation methods, industrial tasks, and worker populations continue to limit direct comparisons and the development of evidence-based recommendations. Furthermore, the extent to which laboratory-based findings translate into reliable, generalizable, and sustained benefits in real-world industrial environments remains insufficiently established.

Accordingly, this systematic review addresses the following questions: RQ1: What types of sensor-integrated technologies and system configurations have been applied to industrial exoskeletons, and what functions do they support?, RQ2: What biomechanical, physiological, ergonomic, and operational outcomes have been reported, and what patterns of effectiveness emerge across different industrial tasks and user populations?, RQ3: What validation methods and performance metrics have been used to assess sensing accuracy, biomechanical estimation, ergonomic risk evaluation, and system reliability?, RQ4: What technological, methodological, human-centered, and operational barriers limit the implementation and generalizability of sensor-integrated exoskeletons in industrial environments?, RQ5: What emerging technological trends and research priorities may support the development of more reliable, adaptive, and human-centered industrial exoskeleton systems? By addressing these questions, the review seeks to integrate the technological and ergonomic evidence while distinguishing reported experimental benefits from the broader requirements for industrial implementation.

Therefore, this systematic review aims to synthesize and critically analyze the scientific evidence on sensor-integrated industrial exoskeleton systems by examining their sensing architectures, industrial applications, reported biomechanical and ergonomic outcomes, validation approaches, implementation challenges, and emerging technological trends, with particular emphasis on their potential contribution to ergonomic risk reduction and the development of reliable, human-centered assistive technologies.

## 2 Methods

### 2.1 Study design and review protocol

This study adopted a structured literature review approach to examine the current state of scientific evidence on industrial exoskeletons for preventing work-related musculoskeletal disorders across industrial environments. To ensure transparency, reproducibility, and a systematic identification and selection of the available evidence, the review process followed the methodological recommendations of the PRISMA 2020 statement (Page et al., 2021). The completed PRISMA checklist is available as (Supplementary Table S1). Furthermore, the research scope and eligibility criteria were conceptually guided by the PECO (Population, Exposure, Comparator, Outcome) framework, providing a structured basis for identifying relevant studies and organizing the qualitative synthesis according to the objectives of the review (Morgan et al., 2018).

### 2.2 PECO framework and research scope

The scope of this review was structured using the PECO (Population, Exposure, Comparator, Outcome) framework to ensure conceptual consistency between the research objective and the evidence selection strategy. The use of the PECO framework is widely recommended for organizing structured literature reviews because it facilitates the clear definition of the research scope, supports transparent study selection, and enhances the reproducibility of the search process (Morgan et al., 2018). Accordingly, the framework was used to define the target population, the exposure of interest, the comparison conditions, and the expected outcomes related to the application of industrial exoskeletons for preventing work-related musculoskeletal disorders. This structured approach also guided the development of the search strategy (Supplementary Table S2), ensuring that the retrieved evidence adequately covered the technological, biomechanical, and implementation dimensions addressed throughout the review (Morgan et al., 2018).

### 2.3 Eligibility criteria

Empirical studies investigating the development, implementation, evaluation, or optimization of industrial exoskeletons in occupational settings were considered eligible, with particular emphasis on research integrating wearable sensing technologies, electromyography, inertial measurement units (IMUs), motion capture, computer vision, biomechanical modeling, and artificial intelligence for ergonomic assessment and work-related musculoskeletal disorder prevention. Eligible studies were required to provide detailed methodological descriptions of the exoskeleton configuration, associated sensing technologies, biomechanical evaluation methods, or validation procedures conducted under real or simulated industrial conditions. Inclusion was restricted to original research articles published in English between 2021 and 2026 and indexed in the Scopus and Web of Science databases.

Taking into account the PECO framework, this eligibility strategy ensured the inclusion of studies addressing industrial applications, biomechanical effectiveness, implementation challenges, and emerging technological developments in industrial exoskeletons. Conversely, review articles, conference papers, editorials, conceptual studies without empirical validation, and research unrelated to occupational applications of industrial exoskeletons were excluded.

### 2.4 Information sources and search strategy

The literature search was conducted in two multidisciplinary scientific databases, Scopus and Web of Science, selected because of their extensive coverage of engineering, industrial systems, occupational ergonomics, and applied technological research. The search was performed on 30 June 2026, and was restricted to peer-reviewed journal articles published between 2021 and 2026. English-language publications were selected using the language filters available in each database. To minimize potential selection bias associated with database-specific restrictions, no open-access filter or additional subject-area restriction was applied during the revised search. These restrictions were applied during the database filtering stage and were recorded separately for each database to ensure transparency and reproducibility.

The search strategy used Boolean operators (AND, OR) to combine terms related to industrial exoskeletons, wearable and biomechanical sensing technologies, electromyography, inertial measurement units, motion capture, computer vision, biomechanical modeling, artificial intelligence, and work-related musculoskeletal disorder. Truncation symbols (*) were applied where appropriate to retrieve lexical variations of the selected terms. The search strategies were developed around the same conceptual domains in both databases, while database-specific syntax and field operators were adapted to the indexing structure of each platform. The initial records corresponded to all number of publications retrieved before the application of the predefined database filters, screening, whereas the final dataset comprised the studies retained after duplicate removal, title and abstract screening, and full-text eligibility assessment and methodological appraisal The complete database-specific search strings are reported below.

Scopus database (“biomechanical sensing” OR “biomechanical monitoring” OR biomechanics OR “human motion analysis” OR “human motion monitoring” OR “wearable sensor*” OR “wearable device*” OR “wearable technology” OR exoskeleton* OR “motion capture”) AND (“industrial ergonomics” OR “occupational ergonomics” OR ergonomics OR “industrial ergonomic assessment” OR “ergonomic risk assessment” OR “occupational risk assessment” OR “musculoskeletal disorder*” OR “work-related musculoskeletal disorder*” OR WMSD*).

Web of Science database (“intelligent biomechanical sensing architecture*” OR “intelligent biomechanical architecture*” OR “biomechanical sensing architecture*” OR “biomechanical sensing system*” OR “biomechanical monitoring system*” OR “biomechanical monitoring” OR “wearable biomechanical system*” OR “wearable sensor*” OR “wearable sensing” OR “smart ergonomic system*” OR “motion capture system*” OR “inertial measurement unit*” OR IMU* OR exoskeleton* OR “sensor fusion” OR “embedded system*” OR “cyber-physical system*” OR “edge computing” OR “artificial intelligence” OR “machine learning” OR “Physics-Informed Neural Network*” OR PINN OR “Extended Kalman Filter” OR EKF OR “ROS 2” OR DDS OR MQTT) AND (“industrial ergonomic assessment” OR “ergonomic risk assessment” OR “industrial ergonomics” OR “occupational ergonomics” OR “musculoskeletal risk assessment” OR “work-related musculoskeletal disorder*” OR WMSD*).

The Boolean search strategies were intentionally developed separately for Scopus and Web of Science rather than using an identical search string in both databases. Preliminary testing showed that applying the Web of Science strategy directly in Scopus substantially reduced the number of retrieved records. Therefore, the search strings were adapted to the indexing characteristics, searchable fields, terminology, and retrieval functionalities of each database to maximize search sensitivity and broaden the identification of potentially relevant studies. The Scopus strategy incorporated broader terms related to biomechanics, wearable sensors, exoskeletons, ergonomics, and musculoskeletal disorders, whereas the Web of Science strategy included additional technological terms related to intelligent biomechanical architectures, sensor fusion, artificial intelligence, machine learning, inertial measurement units, and other emerging technologies.

Despite these differences, both strategies maintained the same core conceptual domains defined by the review question: industrial exoskeletons, biomechanical and wearable sensing technologies, and occupational ergonomic assessment. Thus, the differences between the search strings reflect a database-specific optimization strategy intended to increase the overall breadth and sensitivity of the evidence retrieval rather than a difference in the conceptual scope or eligibility criteria of the review.

### 2.5 Study identification and database filtering

The literature search was conducted in Scopus and Web of Science using database-specific Boolean strategies focused on industrial exoskeletons, biomechanical sensing technologies, wearable systems, and ergonomic assessment. To reduce potential selection bias associated with database-specific restrictions, the revised search did not apply an open-access restriction or an additional Engineering subject-area filter in Scopus. The publication period (2021–2026) and document type (journal articles) were retained in accordance with the predefined eligibility criteria.

The initial search retrieved 8,327 records from Scopus and 409 records from Web of Science, yielding a total of 8,736 records. In Scopus, the publication-year filter (2021–2026) reduced the dataset from 8,327 to 3,135 records, followed by the document-type filter, which retained 1,789 journal articles. In Web of Science, the publication-year filter reduced the initial 409 records to 364, followed by the document-type filter, which retained 316 articles. Consequently, 2,105 records were available for database merging and subsequent study selection.

The records retrieved from Scopus and Web of Science were merged using RStudio. Duplicate detection identified 236 duplicate records, leaving 1,869 unique records for title and abstract screening. During screening, 1,330 records were excluded because they did not meet the predefined PECO eligibility criteria, leaving 539 reports for retrieval. Of these, 372 reports could not be retrieved, resulting in 167 reports available for full-text eligibility assessment.

The full-text eligibility assessment was conducted using the predefined inclusion and exclusion criteria. A total of 61 reports were excluded because they provided insufficient methodological information or reporting (n = 15), insufficient experimental validation (n = 27), or no objective ergonomic outcomes relevant to the review (n = 19). Thus, 106 studies fulfilled all eligibility criteria and were included in the qualitative synthesis.

Importantly, these exclusion criteria represented the final verification of study eligibility and were not equivalent to the subsequent methodological quality or risk-of-bias assessment. The methodological quality and risk of bias of the 106 included studies were assessed separately using the structured qualitative procedure described in Section 2.7. The complete study-selection process is presented in Figure 1.

**FIGURE 1:**
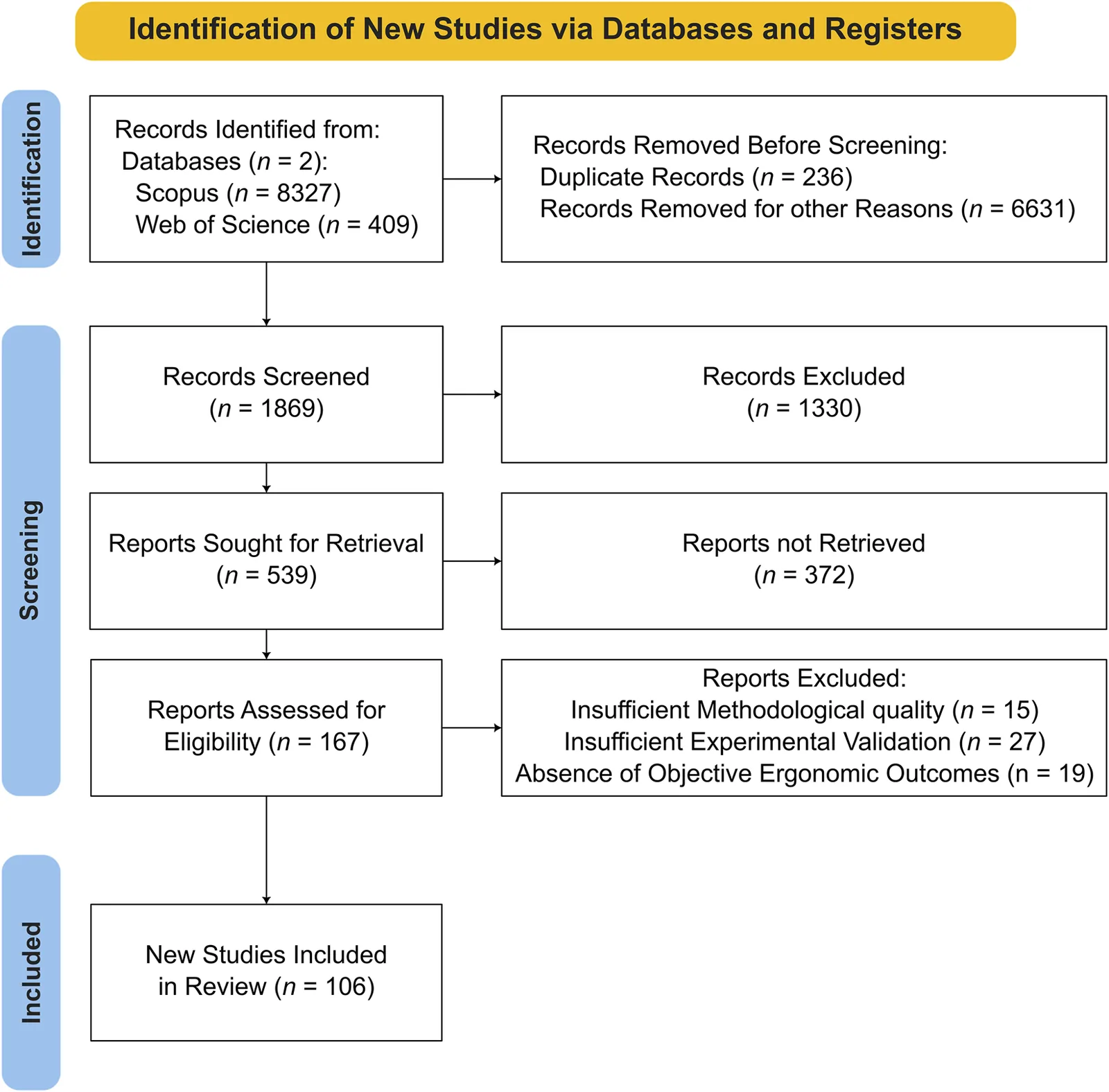
PRISMA 2020 flow diagram illustrating the identification, screening, eligibility assessment, and inclusion of studies. The database search initially identified 8,736 records, comprising 8,327 records from Scopus and 409 from Web of Science. After applying the publication-year and document-type filters, 2,105 records remained (1,789 from Scopus and 316 from Web of Science). Following database merging, 236 duplicate records were removed, leaving 1,869 unique records for title and abstract screening. A total of 1,330 records were excluded during screening, and 539 reports were sought for retrieval. Of these, 372 reports could not be retrieved, leaving 167 reports for full-text eligibility assessment. Sixty-one reports were subsequently excluded because of insufficient methodological information or reporting (n = 15), insufficient experimental validation (n = 27), or absence of objective ergonomic outcomes (n = 19). Ultimately, 106 studies fulfilled all eligibility criteria and were included in the qualitative synthesis.

### 2.6 Study selection and screening procedure

The study selection process followed the sequential stages recommended by the PRISMA 2020 guidelines. The search and consultation of databases were completed on 30 June 2026, and the PRISMA flow diagram was generated using the online visualization tool available at https://estech.shinyapps.io/prisma_flowdiagram/. After duplicate removal, the remaining records entered the screening phase. C.D.R.-Y and A.J.R.-Y independently assessed the titles and abstracts of the retrieved publications according to the predefined PECO eligibility criteria. This stage aimed to determine the relevance of each study with respect to the research objective, specifically focusing on publications addressing the development, implementation, or evaluation of biomechanical monitoring systems and intelligent architectures applied to industrial ergonomic assessment.

Potential disagreements between reviewers were resolved through discussion and consensus. Inter-reviewer agreement during the study-selection process was assessed using Cohen’s kappa coefficient, obtaining κ = 0.81, which indicates substantial agreement. This statistic refers exclusively to the screening and study-selection process and was not used to assess methodological quality or risk of bias.

The full-text eligibility assessment was subsequently conducted for the records considered potentially eligible. The reasons for exclusion at this stage and during the subsequent methodological assessment are presented in the PRISMA flow diagram (Figure 1). The final set of included studies was then subjected to the methodological quality and risk-of-bias assessment described in Section 2.7. The overall workflow of the study-selection procedure is illustrated in Figure 1, which summarizes the stages of identification, screening, eligibility, and final inclusion.

### 2.7 Methodological quality and risk-of-bias assessment

To assess the methodological quality and potential risk of bias of the evidence included in this systematic review, the studies selected following the eligibility and screening stages were evaluated using a structured assessment matrix developed specifically for this review. The assessment considered five domains relevant to the methodological and reporting characteristics of studies on sensor-integrated industrial exoskeletons: (1) Selection, (2) Validity, (3) Setting, (4) Reporting, and (5) Confounding. These domains were selected to capture potential sources of methodological limitations related to participant and scenario selection, measurement and validation procedures, experimental or occupational settings, completeness of methodological reporting, and factors that could affect the interpretation of the reported outcomes.

Each domain was assessed using predefined methodological questions derived from the methodological characteristics expected in empirical studies of industrial exoskeleton systems and their associated sensing technologies. The assessment considered, among other aspects, the clarity of study selection and participant characteristics, the appropriateness and reliability of measurement procedures, the description and relevance of the experimental or occupational setting, the completeness and reproducibility of methodological and outcome reporting, and the identification or control of potential confounding factors. Based on the evidence reported in each study, the overall assessment was classified qualitatively as Low, Some concerns, or High risk of methodological limitations. No external standardized risk-of-bias instrument or numerical composite score was applied, and therefore no numerical cutoff was used to exclude studies on the basis of the quality assessment.

Studies classified as presenting Low risk showed sufficient methodological and reporting information across the assessed domains; Some concerns indicated that one or more domains contained incomplete or unclear information that could affect interpretation; and High indicated substantial methodological or reporting limitations with the potential to affect the reliability or interpretation of the findings. The assessment results were subsequently incorporated into the qualitative synthesis to contextualize the reported evidence regarding ergonomic outcomes, biomechanical effectiveness, sensing technologies, validation approaches, and implementation challenges. The study-selection process was conducted independently by the designated reviewers, whereas the methodological quality and risk-of-bias assessment was performed using the structured assessment matrix described above. This distinction was maintained to ensure that the inter-reviewer agreement reported for the screening process was not interpreted as an agreement statistic for the risk-of-bias assessment.

### 2.8 Data extraction and evidence synthesis

A standardized extraction matrix was used to record bibliographic and study characteristics, including publication year, country, sample size, exoskeleton type and assistance mechanism, occupational task, laboratory or field setting, sensing modality, biomechanical and ergonomic assessment methods, validation procedures, Technology Readiness Level (TRL), and principal quantitative and qualitative outcomes. Multiple outcomes and experimental conditions reported within the same study were retained separately while maintaining the study as the primary unit of analysis. Numerical findings were extracted using the original definitions and measurement units reported by the authors, including percentage changes, electromyographic measures, biomechanical loads, fatigue indicators, classification and prediction performance, intraclass correlation coefficients, and area under the receiver operating characteristic curve. No cross-study statistical normalization was applied to heterogeneous outcome measures, and standardized effect sizes were calculated only when sufficient information and comparable measures were available. The extracted evidence was subsequently synthesized descriptively according to exoskeleton type, sensing modality, occupational task, study setting, and principal ergonomic outcome.

### 2.9 Bibliometric analysis

Bibliographic records retrieved from Scopus and Web of Science were processed in RStudio for duplicate identification and removal and subsequently analyzed using VOSviewer. Bibliographic data were imported using the “Read data from bibliographic database” option, and bibliometric networks were constructed to examine keyword co-occurrence, author relationships, and country citation patterns, together with an overlay visualization to explore the temporal evolution of the identified research themes. Full counting was selected as the counting method, with a minimum occurrence threshold of five occurrences for keywords. Network normalization was performed using association strength, with a clustering resolution of 1.00, a minimum cluster size of 1, and a scale value of 1. The occurrence attribute was used as the weighting criterion for keyword analysis. The resulting networks were interpreted descriptively to identify thematic clusters, patterns of scientific collaboration, geographical citation relationships, and emerging research topics related to sensor-integrated industrial exoskeletons.

### 2.10 Data synthesis

Because of substantial methodological and clinical heterogeneity in exoskeleton designs, sensing configurations, participant characteristics, occupational tasks, experimental conditions, and outcome measures, no meta-analysis was performed. The evidence was therefore synthesized qualitatively and supported by descriptive quantitative reporting of study-level findings, preserving the original outcome metrics and measurement units. Results were organized according to exoskeleton type, sensing modality, occupational task, study setting, and principal ergonomic outcome, with laboratory and field investigations distinguished to facilitate interpretation of experimental validation and occupational applicability. This approach enabled the characterization of technological configurations, ergonomic outcomes, validation approaches, implementation challenges, and emerging trends without assuming a common effect size across heterogeneous studies.

## 3 Results

### 3.1 Characteristics of included studies

The included studies showed considerable heterogeneity in terms of occupational domains, targeted anatomical regions, mechanical actuation architectures, sensing technologies, and deployment settings. Most evaluations were conducted in simulated laboratory environments (TRL 4–5), with a strong representation of industrial, logistics, and manufacturing contexts. The evidence covered a wide range of sectors, including logistics, warehousing, manufacturing, automotive assembly, construction, general ergonomics assessment, healthcare, and agro-industry.

The characteristics of the included studies are summarized in Table 1, which presents the main characteristics of the scientific literature ( n=106 ), detailing the main occupational domain, target body regions, performance design, detection systems and the Technology Readiness Level (TRL). The studies are organized by occupational domain—led by logistics and materials handling (29.2%), manufacturing and automotive (23.6%), and construction (16.0%)—and are further grouped by mechanical actuation architecture (such as passive elastic/spring systems, active motors, soft robotics, and non-exoskeleton postural assessment systems) to improve clarity and facilitate comparison between technological solutions.

**TABLE 1:** Exoskeleton and sensor system characteristics across occupational domains (*n* = 106).

Occupational domain and sector	*n* (%/106)	Targeted anatomical region	Mechanical actuation architecture	Sensing & sensor fusion systems	Technology readiness level (TRL) and setting	Author (Year)
Logistics, warehousing and material handling	31 (29.2%)	Trunk/Lumbar region (L5–S1); lower limbs	Passive elastic (64.5%); active/Powered (29.0%); hybrid (6.5%)	Multimodal fusion: Muscle sEMG + insole Pressure/FSR + trunk IMUs	- TRL 4–5 (74.2% simulated Lab) - TRL 6–7 (25.8% field)	Matijevich et al. (2021); Wang et al. (2025); Adhaye et al. (2026); Jakobsen et al. (2023); Rodzak et al. (2024); Gutiérrez et al. (2024); Zhu et al. (2024); Slaughter et al. (2025); Van Sluijs et al. (2024); Schubert and Weidner (2025); Le and Lin (2026); Park et al. (2023); Nail-Ulloa et al. (2024); Ramella et al. (2024); Thevenot et al. (2026); Cuttilan et al. (2023); Jakobsen et al. (2024); Bhardwaj et al. (2023); Correa et al. (2023); Yan et al. (2024); Mitterlehner et al. (2023); Callihan et al. (2023); Donisi et al. (2023); Umar et al. (2021); Michaud et al. (2023); Liu et al. (2026); Satpute et al. (2024); Rinaldi et al. (2026); Taori and Lim (2024); Hubaut et al. (2022); Forgione et al. (2026)
Manufacturing, assembly and automotive	25 (23.6%)	Upper limbs/Shoulder (overhead work); trunk	Passive spring/gas (52.0%); active motor (36.0%); assessment systems (12.0%)	Multichannel sEMG + inertial IMUs + computer vision (YOLOv8/Azure Kinect)	TRL 4–5 (68.0% Lab); TRL 6–7 (32.0% industrial plant)	Dimo et al. (2024); Morillo et al. (2026a) Morillo et al. (2026b); Refai et al. (2025); Scotto Di Luzio et al. (2025); Coccia et al. (2024); Monaco et al. (2023); Razavi et al. (2024); Krishnan et al. (2025); Tu et al. (2022); Caporaso et al. (2022); Wang et al. (2024); Vora et al. (2026); Kazemi and Srinivasan (2026); Simon et al. (2024); Ofori et al. (2025); Musso et al. (2024); Papetti et al. (2025); Matos et al. (2024); Terlizzi et al. (2025); Donisi et al. (2021); Jia et al. (2026); Laffranchi et al. (2021); Ascari et al. (2021); Wu et al. (2025)
Construction, Civil works and infrastructure	17 (16.0%)	Trunk; lower limbs (Stooping/Squatting); multi-joint	Passive support (58.8%); non-exoskeleton posture monitoring (41.2%)	Vision markerless motion capture + IMUs + Goniometry/FBG fibers	TRL 3–4 (52.9% Prototype); TRL 6–7 (47.1% on-site field)	Al-Khiami and Lindhard (2026a); Seo et al. (2023); Albalawi (2026); Zelik et al. (2022); Trkov et al. (2022); Chen et al. (2025); Jakobsen et al. (2025); Madinei and Nussbaum (2023); Van Sluijs et al. (2023); Al-Khiami et al. (2026); Skals et al. (2021); Oh et al. (2025); Ojha et al. (2024); Ojha et al. (2025); Lim and Yang (2023); Brandt et al. (2025); Ko et al. (2024)
General ergonomics and occupational assessment	16 (15.1%)	Whole-body/Multi-joint postural assessment	Sensorized ergonomic risk assessment systems	Inertial fusion (IMUs) + automated RULA/REBA algorithms + bio-sensors	TRL 3–5 (87.5% concept proof and controlled lab)	Di Natali et al. (2021); Tanthuwapathom et al. (2026); Young et al. (2021); Vox et al. (2021); Panariello et al. (2022); Maksimović et al. (2022); Sridhar et al. (2025); Yin et al. (2021); Shim et al. (2023); Kuber and Rashedi (2024); Concha-Pérez et al. (2023); Moyen-Sylvestre et al. (2022); Agostinelli et al. (2021); Tanthuwapathom et al. (2025); Blanco et al. (2022); Aqueveque et al. (2023)
Occupational health, rehabilitation and care	13 (12.3%)	Trunk; shoulder & wrist	Soft active robotics (46.2%); passive (53.8%)	High-density sEMG + interface FSR sensors + Strain sensors	TRL 4–5 (76.9% Lab biomechanical trials)	Öçal et al. (2023); Ghonasgi et al. (2021); Zhang and Huang (2024); Tahir et al. (2023); Brunner et al. (2022); Brunner et al. (2023); Choi et al. (2026); Kong et al. (2023); Kong et al. (2022); Gholami et al. (2022); Panero et al. (2025); Tomezzoli et al. (2026); Pratapneni et al. (2026)
Agro-industry, harvesting and farming	4 (3.8%)	Lumbar region; shoulders (high-reach harvesting)	Structural passive (75.0%); active (25.0%)	Waterproof IMUs + portable sEMG + physiological monitoring	TRL 5–6 (100% agricultural field trials)	Rossini et al. (2021); Kong et al. (2021); Cividino et al. (2024); Muslim et al. (2025)

Most of the included empirical evaluations were controlled laboratory experiments under simulated tasks, with a smaller proportion of field or *in situ* trials (25.8% in logistics, 32.0% in manufacturing, and 47.1% in construction), and only agricultural studies showing full field deployment (100%). This distribution reflects the emerging nature of research in this field and highlights the predominance of controlled experimental settings over real-world industrial evaluations.

To clarify the conceptual scope of the review, Table 1 distinguishes between industrial exoskeleton systems and complementary sensor-based ergonomic assessment technologies. Exoskeleton systems were considered the primary assistive technologies when passive, active, hybrid, or soft robotic structures were used to provide physical support or assistance to workers. In these systems, sensing technologies such as surface electromyography (sEMG), inertial measurement units (IMUs), force-sensitive resistors (FSRs), physiological sensors, motion capture, and computer vision were considered integrated components for biomechanical monitoring, ergonomic assessment, or human–exoskeleton interaction.

In contrast, studies involving standalone posture-monitoring or sensorized ergonomic assessment systems without an exoskeleton were classified as complementary assessment technologies. These systems do not provide mechanical assistance and therefore were not considered exoskeletons or interpreted as evidence of exoskeleton effectiveness. Their inclusion provides contextual evidence on sensing architectures and biomechanical assessment methods relevant to the design, validation, and evaluation of sensor-integrated industrial exoskeletons.

### 3.2 Sensor integration in industrial exoskeleton systems

Surface electromyography (sEMG/EMG) and inertial measurement units (IMUs) were the sensing technologies most frequently integrated into industrial exoskeleton systems (Figure 2). These sensors were combined with motion capture systems, pressure sensors, physiological sensors, and optical tracking technologies to quantify muscle activation, joint kinematics, lumbar loading, trunk inclination, range of motion, fatigue, and ergonomic risk during occupational tasks (Ghonasgi et al., 2021; Iranzo et al., 2022; Kong et al., 2022; Gutiérrez et al., 2024; Simon et al., 2024). Reported quantitative outcomes included posture assessment with intraclass correlation coefficients ranging from 0.89 to 0.92 (Terlizzi et al., 2025), lumbar load estimation with $R^{2}=0.89$ (Morillo et al., 2026b), and biomechanical measurements showing a bias lower than 1° compared with the VICON motion capture system (Hubaut et al., 2022) (Supplementary Table S3).

**FIGURE 2:**
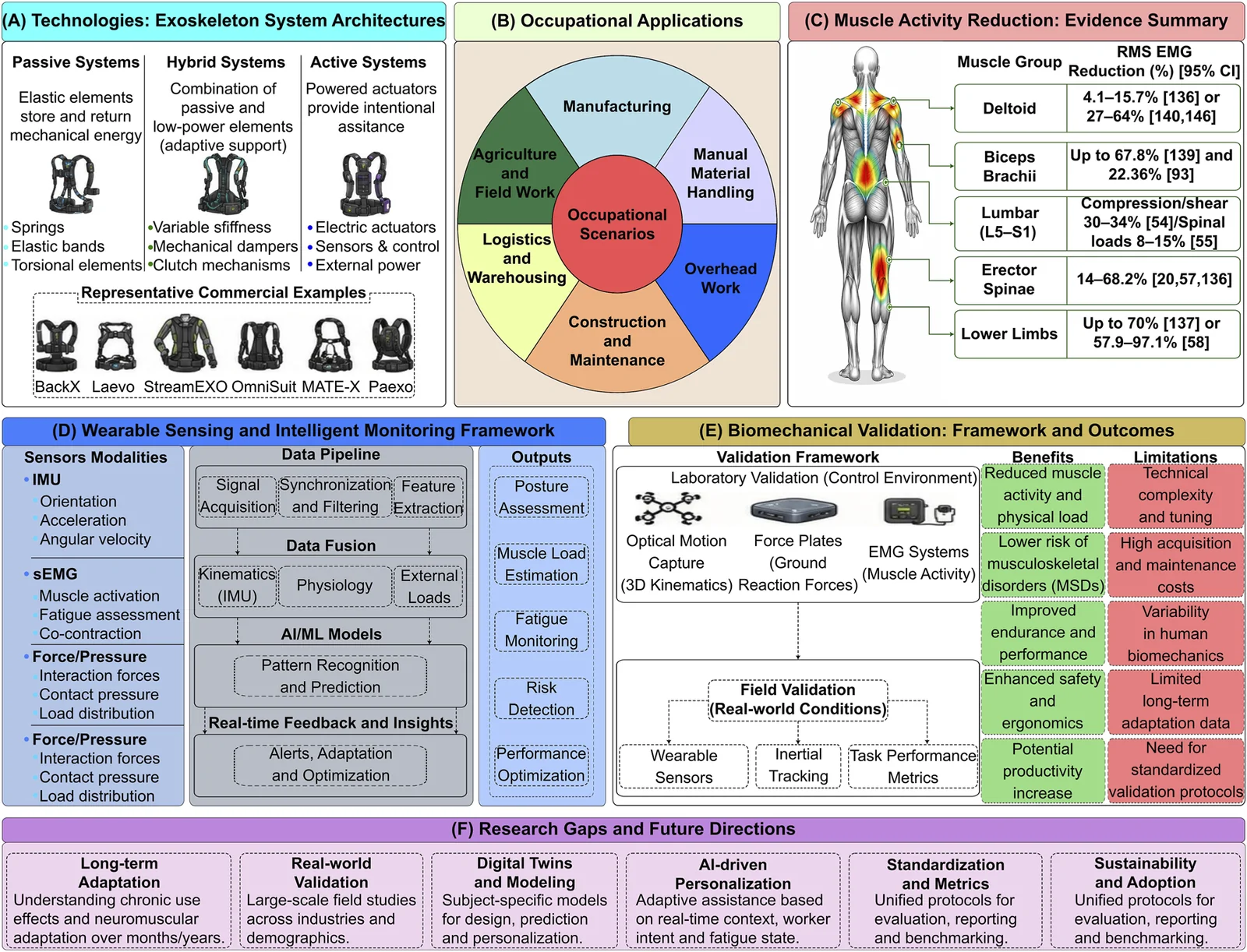
Integrated holistic framework of passive, hybrid, and active exoskeleton technologies based on the systematic review (*N* = 106 studies, 2021–2026). **(A)** Classification of mechanical assistance systems and representative commercial devices; **(B)** Distribution of occupational application scenarios identified in the literature; **(C)** Quantitative evidence summary of muscle activity reduction (% RMS EMG) across main muscle groups; **(D)** Wearable sensing, data fusion pipelines, and machine learning monitoring framework; **(E)** Methodological validation spectrum (laboratory vs. field conditions) along with identified benefits and technical limitations; **(F)** Key research gaps and future strategic directions for Industry 5.0 integration. CI: Confidence Interval; MSDs: Musculoskeletal Disorders; RMS: Root Mean Square; sEMG: Surface Electromyography; IMU: Inertial Measurement Unit.

Sensor-based monitoring platforms also incorporated computational algorithms for automated biomechanical assessment. Wearable electromyography combined with support vector machines reported a ROC of 0.985 for biomechanical risk classification (Tanthuwapathom et al., 2025), whereas semi-automatic IMU platforms based on the NIOSH lifting equation achieved an ergonomic assessment accuracy of 98.5% (Razavi et al., 2024). Inertial motion capture systems reported F1 scores of 0.97 for risk classification and 1.00 for no-risk classification (Govaerts et al., 2023), while machine vision systems achieved an average ICC of 0.967 for posture estimation (Kong et al., 2022). Additionally, wearable EMG devices identified lifting and lowering events with accuracies of 86.7% and classified handled loads with an accuracy of 80.9% (Wang et al., 2021).

### 3.3 Industrial applications of sensor-integrated exoskeletons

Sensor-integrated exoskeletons were evaluated during manual material handling, repetitive lifting, overhead work, assembly, construction, logistics, manufacturing, agriculture, and office activities (Gholami et al., 2022; Cividino et al., 2024; Ofori et al., 2025; Kazemi and Srinivasan, 2026). Across these occupational scenarios, the reported biomechanical outcomes included reductions in muscle activation ranging from 8% to 70% (Cuttilan et al., 2023; Madinei and Nussbaum, 2023; Shim et al., 2023; van Sluijs et al., 2023; Kuber and Rashedi, 2024; Musso et al., 2024; Ramella et al., 2024; Thevenot et al., 2026), decreases in lumbar compression and shear forces of 8%–34% (Madinei and Nussbaum, 2023; Nail-Ulloa et al., 2024; Thevenot et al., 2026), reductions in muscle fatigue of up to 60% (Ojha et al., 2024), and improvements in postural stability of 24.8%–35.4% during material handling tasks (Oh et al., 2025). Studies also reported reductions in perceived physical effort and ergonomic risk during repetitive and physically demanding activities (Ojha et al., 2024; Kazemi and Srinivasan, 2026; Thevenot et al., 2026).

Industrial applications also demonstrated improvements in operational performance through sensor-based monitoring. Productivity increased between 15.3% and 59.5% in construction-related activities (Ofori et al., 2025; Oh et al., 2025), while metabolic cost decreased by 17% during manual handling tasks using active lumbar exoskeletons (Ojha et al., 2024). Intelligent monitoring systems achieved classification accuracies of 95.7% for physical effort detection (Concha-Pérez et al., 2023) and 99% for identifying experimental conditions based on electromyographic signals (Vora et al., 2026). These applications were evaluated in both laboratory protocols and real industrial environments involving manufacturing, logistics, construction, supermarkets, agriculture, and office work (Skals et al., 2021; Mitterlehner et al., 2023; Adhaye et al., 2026; Kazemi and Srinivasan, 2026).

### 3.4 Biomechanical and ergonomic outcomes

The reviewed studies consistently reported improvements in biomechanical outcomes following the implementation of sensor-integrated exoskeleton systems. Muscle activity was the most frequently evaluated variable, with electromyographic reductions ranging from 13% to 68.2% across the deltoid, trapezius, erector spinae, biceps, triceps, and lower-limb muscles during manual handling, overhead work, repetitive lifting, and assembly operations (Brunner et al., 2023; Park et al., 2023; Musso et al., 2024; Forgione et al., 2026; Thevenot et al., 2026). Lumbar biomechanical loading also decreased substantially, with reductions of 8%–34% in spinal compression and shear forces (Madinei and Nussbaum, 2023; Nail-Ulloa et al., 2024), 10%–27% in lumbar loading during logistics tasks (Vatan et al., 2025), and decreases of up to 93% in cumulative back damage estimated through fatigue-based ergonomic models (Maksimović et al., 2022). Postural outcomes included reductions in trunk flexion, improved movement stability, and maintenance of natural joint kinematics without significant movement restrictions during lifting and repetitive occupational activities (Laffranchi et al., 2021; Blanco et al., 2022; van Sluijs et al., 2023; Oh et al., 2025).

Ergonomic performance was also quantified through indicators of fatigue, physical demand, and occupational risk. Muscle fatigue decreased by up to 60%, while metabolic cost was reduced by 17%, oxygen consumption by more than 24%, cardiac cost by 15%, and perceived physical exertion by 21.5% during physically demanding tasks (Park et al., 2023; Ojha et al., 2024; Taori and Lim, 2024). Intelligent ergonomic assessment systems reported ROC values of 0.985 for biomechanical risk prediction (Tanthuwapathom et al., 2025), 98.5% accuracy for automated ergonomic assessment (Razavi et al., 2024), F1 scores between 0.97 and 1.00 for occupational risk classification (Govaerts et al., 2023), and posture estimation with an average ICC of 0.967 (Kong et al., 2022). Additionally, construction and industrial studies documented productivity improvements ranging from 15.3% to 59.5%, together with reductions in ergonomic exposure during manual material handling and repetitive work activities (Ofori et al., 2025; Oh et al., 2025).

### 3.5 Intelligent monitoring and data processing strategies

The included studies incorporated intelligent data-processing strategies by integrating wearable sensing technologies with computational algorithms for automated biomechanical assessment. Surface electromyography, inertial measurement units, motion capture systems, RGB cameras, pressure sensors, smartwatches, eye-tracking devices, LiDAR sensors, and physiological monitoring platforms were combined to acquire synchronized kinematic, physiological, and ergonomic data during occupational activities (Matijevich et al., 2021; Madinei et al., 2022; Wu et al., 2025; Choi et al., 2026; Tanthuwapathom et al., 2026). Several monitoring systems also integrated multimodal sensor fusion, allowing the simultaneous evaluation of posture, muscle activity, movement patterns, physiological responses, and ergonomic exposure within a single acquisition platform (Madinei et al., 2022; Tanthuwapathom et al., 2026).

Computational processing was primarily based on machine learning, deep learning, computer vision, and biomechanical modelling. Support vector machines achieved a ROC of 0.985 for biomechanical risk prediction using wearable electromyography (Tanthuwapathom et al., 2025), whereas gradient-boosted decision trees estimated lumbar loading with $R^{2}=0.89$ using trunk IMUs and instrumented insoles (Morillo et al., 2026b). Convolutional neural networks classified manual lifting risk with an accuracy of 89% by combining pressure-sensitive gloves and machine vision (Chen et al., 2025), while automatic task-recognition algorithms identified manual handling activities with accuracies of 85.3% using pressure sensors and accelerometers integrated into instrumented insoles (Monaco et al., 2023). Single-camera vision-based deep learning models reported an $r^{2}$ of 0.939 for posture estimation (Afolabi et al., 2025), and multimodal physiological monitoring using smartwatches improved fatigue classification from F1=0.8848 to 0.9375 for binary classification and from 0.8232 to 0.8793 for four fatigue levels (Morillo et al., 2026b).

### 3.6 Current challenges for industrial implementation

The most frequently reported limitations included the need for validation under real working conditions, larger and more heterogeneous study populations, and long-term evaluations to verify system performance during complete work shifts (Donisi et al., 2023; Tahir et al., 2023; Schubert and Weidner, 2025; Scotto Di Luzio et al., 2025; Forgione et al., 2026). User-related factors were also recurrently documented, including the need for anthropometric adaptation, worker training, familiarization processes, and acceptance to ensure effective integration into occupational activities (Öçal et al., 2023; Coccia et al., 2024; Rodzak et al., 2024; Jakobsen et al., 2025; Liu et al., 2026). Additionally, some systems reported restricted performance according to task characteristics, posture, or operating conditions, limiting their applicability across different industrial scenarios (Jakobsen et al., 2023; Michaud et al., 2023).

Technical and organizational challenges were also reported for intelligent monitoring platforms. Several studies indicated the need for periodic sensor calibration, synchronization of multimodal acquisition systems, and specialized infrastructure for motion capture, virtual reality, LiDAR, or robotic assistance, increasing implementation complexity and operational costs (Ascari et al., 2021; Caporaso et al., 2022; Aqueveque et al., 2023; Jakobsen et al., 2025; Wang et al., 2025). Other reported constraints included interoperability between heterogeneous sensing devices, dependence on computational processing for real-time analysis, and the requirement for standardized validation protocols before large-scale industrial deployment (Umar et al., 2021; Trkov et al., 2022; Al-Khiami et al., 2025; Albalawi, 2026).

The methodological quality appraisal revealed high compliance in experimental protocol definition (96.2%) and sensor specifications (89.6%). However, critical gaps were identified in ecological validity, as only 15.1% of studies were performed in real industrial settings and 8.5% involved full-shift longitudinal evaluations (Supplementary Table S4).

### 3.7 Emerging trends and research opportunities

The reviewed literature showed a progressive transition from single-sensor configurations toward multimodal monitoring systems integrating electromyography, inertial measurement units, motion capture, pressure sensors, physiological monitoring, computer vision, LiDAR, and wearable devices within unified acquisition platforms (Agostinelli et al., 2024; Jia et al., 2026; Pratapneni et al., 2026). Several studies also incorporated artificial intelligence techniques, including support vector machines, convolutional neural networks, gradient-boosted decision trees, and deep learning algorithms, to automate biomechanical risk assessment, task recognition, posture estimation, and fatigue monitoring under occupational conditions (Yan et al., 2021; Agostinelli et al., 2026). These developments were accompanied by increasing implementation of portable and wearable monitoring architectures capable of continuous biomechanical data acquisition during industrial activities (Schiebl et al., 2022; Zhang and Huang, 2024).

Recent studies also expanded the integration of computational biomechanical models with intelligent sensing technologies. Musculoskeletal simulations, inverse kinematic models, digital human modelling, and machine vision systems were combined with wearable sensors to quantify biomechanical exposure and support objective ergonomic assessment in manufacturing, logistics, construction, and manual material handling tasks (Panariello et al., 2022; Zelik et al., 2022; Dimo et al., 2024). This thematic group is dominated by the terms ergonomics, wearable devices, wearable electronic devices, Industry 5.0, machine learning, human-machine systems, and risk assessment (Figure 3). These concepts indicate that research is increasingly focused on integrating exoskeletons into smart manufacturing environments using digital technologies capable of supporting ergonomic decision-making, continuous monitoring, and the prevention of occupational hazards.

**FIGURE 3:**
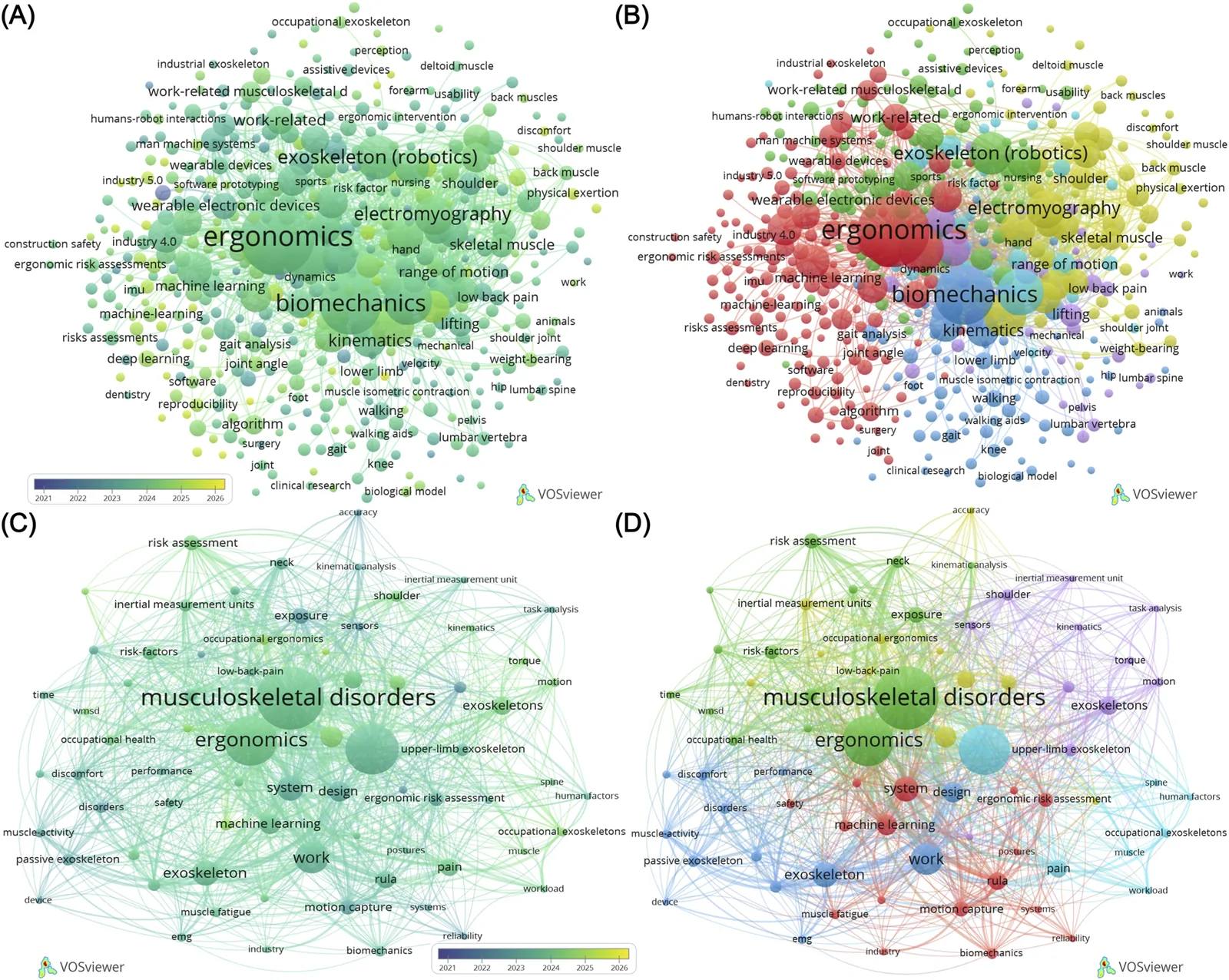
Bibliometric co-word network architectures and temporal evolutionary trends of industrial implementation challenges. The upper panels represent the primary ergonomic-biomechanical re-search cluster under **(A)** temporal overlay visualization (gradient timeline from 2021 to 2026) and **(B)** discrete network cluster visualization mapping interacting thematic domains. The lower panels illustrate the broader clinical-systemic interaction network focused on work-related musculoskeletal disorders, displayed via **(C)** evolutionary overlay chronology and **(D)** multi-cluster thematic modularity. Node sizes are proportional to the absolute keyword co-occurrence frequency (*N*), while the link thickness indicates the association strength based on the normalized association strength index.

## 4 Discussion

### 4.1 Principal findings

The reviewed evidence demonstrates that sensor-integrated exoskeleton systems can reduce biomechanical exposure during physically demanding industrial activities, with reported decreases in muscle activation ranging from 13% to 68.2% and reductions in spinal loading between 8% and 34% depending on the assisted body region, exoskeleton configuration, occupational task, and assessment method (Kong et al., 2022; Madinei and Nussbaum, 2023; Terlizzi et al., 2025; Le and Lin, 2026). However, these percentages should be interpreted as study-specific findings rather than as pooled or generalizable effect estimates. The included studies differed substantially in sample size, assistance mechanism, sensor configuration, participant characteristics, task demands, experimental conditions, and outcome definitions, which prevented meaningful quantitative pooling. Several investigations also used relatively small samples or controlled laboratory protocols, limiting the extent to which the reported reductions can be extrapolated to prolonged industrial work (Iranzo et al., 2022; Mitterlehner et al., 2023; Jakobsen et al., 2025). Furthermore, selective publication or reporting of favorable outcomes cannot be excluded given the heterogeneity of the available evidence. Therefore, the magnitude of the reported reductions should be considered within the methodological and contextual characteristics of each study rather than interpreted as a uniform effect of sensor-integrated exoskeleton systems.

The effects observed in the physical activities of operators are associated with the ability of exoskeletons to provide external mechanical support while simultaneously monitoring biomechanical responses using wearable sensors, such as electromyography sensors and inertial measurement units (Govaerts et al., 2023; Sridhar et al., 2025). The incorporation of artificial intelligence and multimodal sensing has further expanded the role of exoskeletons from assistive devices toward intelligent ergonomic assessment platforms. Machine learning models achieved ROC values of 0.985 for biomechanical risk classification and R^2^ values of 0.89 for lumbar load estimation, indicating the potential of data-driven approaches for automated occupational risk evaluation (Matijevich et al., 2021; Donisi et al., 2023). These improvements are explained by the integration of physiological, kinematic, and contextual information, which enables more comprehensive exposure characterization compared with isolated ergonomic assessments (Ciullo et al., 2021; Seo et al., 2023).

### 4.2 Technological advances in sensor-integrated exoskeleton systems

The technological evolution of sensor-integrated exoskeleton systems is characterized by the transition from laboratory-based biomechanical evaluation toward portable and intelligent monitoring architectures. Recent developments demonstrated that inertial-based ergonomic systems can achieve strong agreement with conventional assessments, with posture estimation showing intraclass correlation coefficients between 0.89 and 0.92 compared with RULA-based evaluations (Tanthuwapathom et al., 2025). Similarly, wearable motion devices and 3D-printed sensing solutions reported high measurement reliability, achieving determination coefficients of 0.991 for flexion–extension and 0.972 for radial–ulnar deviation estimation (Young et al., 2021). These advances indicate that miniaturization and portability are improving the feasibility of continuous ergonomic monitoring (Jakobsen et al., 2024; Refai et al., 2025). However, as demonstrated in our quality assessment (Supplementary Table S3), these findings highlight that despite high sensor accuracy, the field suffers from a lack of ecological validity; reliance on short laboratory trials with healthy surrogates limits the generalizability of current industrial exoskeletons to actual shift-long workplace operations.

A further technological progression involves the incorporation of artificial intelligence and automated biomechanical interpretation, which enables exoskeleton systems to move beyond data acquisition toward real-time decision support. Machine vision approaches demonstrated reliable posture reconstruction, with average ICC values of 0.967 and angular estimation errors between 2.10° and 10.98° using single-camera deep learning systems (Pratapneni et al., 2026). Likewise, automatic ergonomic assessment platforms based on optical and inertial sensing achieved approximately 98.5% accuracy when digitizing the NIOSH lifting assessment methodology (Gutiérrez et al., 2024). These findings suggest that intelligent processing can reduce evaluator dependency and improve scalability; nevertheless, the adoption of these systems is still restricted by computational requirements, sensor synchronization, and the need for standardized validation in heterogeneous industrial environments (Musso et al., 2024; Zhu et al., 2024; Al-Khiami et al., 2025).

### 4.3 Implications for ergonomic risk reduction

The reviewed evidence indicates that sensor-integrated exoskeleton systems can strengthen musculoskeletal disorder prevention by enabling continuous identification of biomechanical exposure factors during occupational activities. Unlike conventional observational methods, wearable sensing platforms quantify dynamic risk conditions through posture, muscle activity, movement variability, and physiological responses (Correa et al., 2023; Panero et al., 2025; Al-Khiami and Lindhard, 2026b; Al-Khiami et al., 2026). For example, sensor-based ergonomic monitoring identified work-related asymmetries not detected by traditional assessments, showing significant differences in fatigue progression throughout work periods (*F* (1,17) = 22.402; p < 0.001) (Simon et al., 2024). Similarly, intelligent assessment systems combining IMU, EMG, and ergonomic models improved the characterization of individual exposure patterns, supporting more personalized preventive interventions (Weston et al., 2022; Papetti et al., 2025; Vora et al., 2026).

The contribution of these technologies extends beyond risk detection by supporting safer and more efficient occupational performance through real-time feedback and adaptive assistance. Construction and industrial studies demonstrated that exoskeleton-supported activities improved productivity between 15.3% and 59.5%, while robotic assistance systems reduced task execution time by 59.46% during collaborative operations (Oh et al., 2025; Wu et al., 2025). Additional wearable monitoring approaches, including smartwatches, instrumented insoles, and physiological sensing platforms, enhanced fatigue recognition and task classification, improving the capacity to identify hazardous behaviors before excessive exposure occurs (Marín-Boné and Marin, 2021; Al-Khiami et al., 2026). However, successful implementation requires considering worker acceptance, anthropometric adaptation, training, and operational compatibility, since field evaluations reported differences between laboratory effectiveness and real workplace performance (Yin et al., 2021; Tu et al., 2022; Satpute et al., 2024).

### 4.4 Barriers to industrial translation

Despite the demonstrated potential of sensor-integrated exoskeletons, their industrial translation remains limited by insufficient validation under real working conditions and restricted participant diversity. Several studies reported evaluations with small samples, including agricultural monitoring systems tested with only four workers and wrist-assessment technologies validated with six participants, reducing confidence in cross-sector generalization (Cividino et al., 2024; Tomezzoli et al., 2026). Furthermore, marker less motion capture systems showed limitations in complex postures, with trunk rotation estimation errors reaching 14.04°, indicating sensitivity to movement occlusion and task variability (Brunner et al., 2022; Wang et al., 2024).

Technical and organizational constraints also restrict large-scale adoption because intelligent ergonomic systems frequently require complex infrastructures, specialized personnel, and continuous calibration. Virtual reality-based ergonomic platforms reported angular deviations between ±6° and ±42° compared with reference systems, while LiDAR-based assessment frameworks required advanced sensing infrastructure and computational processing capabilities (Vox et al., 2021). Similarly, multimodal monitoring platforms integrating physiological, kinematic, and environmental information require synchronization of multiple devices, increasing implementation complexity (Bhardwaj et al., 2023; Grazi et al., 2024; Rinaldi et al., 2026). Additionally, worker acceptance and adaptation remain critical, as field studies identified differences in usability and effectiveness between laboratory and industrial conditions (Carbonaro et al., 2021; Donisi et al., 2021; Humadi et al., 2021; Zhou and Zheng, 2021).

### 4.5 Future perspectives for intelligent industrial exoskeletons

Future intelligent industrial exoskeletons should evolve from monitoring-oriented platforms toward adaptive cyber-physical systems capable of integrating real-time sensing, predictive models, and personalized assistance strategies (Rossini et al., 2021; Afolabi et al., 2024; Krishnan et al., 2025). Current evidence already demonstrates the feasibility of this transition through multimodal architectures combining EMG, IMU, physiological sensors, and environmental data, such as platforms that simultaneously evaluate ergonomic and organizational factors during industrial operations (Kong et al., 2021; Jakobsen et al., 2024; Wu et al., 2025). Furthermore, individualized biomechanical models integrated with sensor measurements have improved worker-specific exposure estimation during lifting tasks (Schwerha et al., 2022), while digital human models have enabled the simultaneous analysis of kinematic, kinetic, and muscular variables during occupational activities (Brunner et al., 2022). These advances support the future integration of Digital Twins and Physics-Informed Neural Networks to continuously update worker models and predict biomechanical overload (Manivasagam and Yang, 2022; Govaerts et al., 2024).

The next-generation of exoskeletons will likely incorporate Edge AI and Cyber-Physical Systems to achieve faster and more autonomous ergonomic decision-making (Marín-Boné and Marin, 2021; Moyen-Sylvestre et al., 2022; Yan et al., 2024). Current intelligent platforms demonstrate this potential through automated task recognition using pressure sensors and accelerometers, reaching accuracies of 85.3%, and through wearable EMG systems capable of identifying lifting and lowering activities with accuracies of 86.7% and load classification of 80.9% (Laffranchi et al., 2021; van Sluijs et al., 2024). Similarly, AI-based fatigue monitoring using smartwatch-derived physiological variables improved classification performance from F1 = 0.8848 to 0.9375 for binary fatigue detection (Shakourisalim et al., 2024; Slaughter et al., 2025; Morillo et al., 2026a). These results indicate that decentralized AI processing could enable adaptive assistance and early risk prediction; however, future developments require larger industrial datasets, interoperability standards, and validation under diverse workplace conditions (Di Natali et al., 2021; Bonakdar et al., 2025; Terlizzi et al., 2025).

### 4.6 Evidence gaps and research priorities for industrial exoskeleton systems

The current evidence indicates that the main research gap is the limited confirmation of long-term effectiveness under real industrial conditions (Lim and Yang, 2023; Ojha et al., 2025; Hong et al., 2026). Although several systems achieved high technical performance, many validations remain restricted to small experimental samples, including studies with 4 agricultural workers, 6 industrial participants, and fewer than 20 subjects in multiple exoskeleton evaluations (Agostinelli et al., 2021; Ding et al., 2023; Cividino et al., 2024; Matos et al., 2024). Likewise, several technologies reported high accuracy values, such as ergonomic classification above 95% or biomechanical estimation with R^2^ values close to 0.89, but these outcomes were mainly obtained under controlled protocols rather than complete work shifts (Matijevich et al., 2021; Vox et al., 2021; Orhan et al., 2025). Therefore, future research should prioritize longitudinal industrial trials, larger heterogeneous populations, and standardized evaluation frameworks to verify sustained ergonomic benefits (Onofrejová et al., 2022; Callihan et al., 2023; Mitterlehner et al., 2023).

### 4.7 Adverse effects, usability, acceptance, and implementation considerations

Previous reviews and empirical studies indicate that the benefits of occupational exoskeletons must be interpreted with due regard for potential adverse effects, usability limitations, and worker acceptance. The considerable heterogeneity in the application of occupational exoskeletons across the healthcare, social care, and industrial sectors, highlighting differences in devices, tasks, outcomes, and implementation contexts, underscores the complexity of the implementation process (Bhat et al., 2025). This heterogeneity is also evident in the present review and reinforces the need to avoid interpreting biomechanical reductions as sufficient evidence of overall efficacy. In particular, reductions in muscle activation or spinal loading do not necessarily imply a uniform decrease in physical demands across the entire musculoskeletal system.

Potential unintended biomechanical consequences warrant special attention, as studies on lumbar and upper-limb support exoskeletons have reported changes in trunk movement, plantar pressure, muscle activation, and biomechanical loading in body regions not directly assisted by the device (Wang et al., 2024; Grazi et al., 2024; Hong et al., 2026). Changes in torso movement and dynamic plantar pressure were observed when using a lumbar support exoskeleton during asymmetric load handling (Wang et al., 2024). Similarly, it has been shown that an upper-limb exoskeleton could reduce shoulder muscle load while simultaneously influencing metabolic cost, highlighting that the benefits of assistance may depend on the specific task and the biomechanical strategy employed (Hong et al., 2026). These findings suggest that the evaluation of exoskeletons should not focus exclusively on the target muscle group or joint but must also examine the potential redistribution of mechanical demands across adjacent anatomical regions.

Usability, comfort, and worker acceptance represent additional dimensions of implementation. Field research on passive shoulder exoskeletons has evaluated biomechanical changes together with acceptance and usability during manual material handling, demonstrating the importance of assessing the interaction between the device and actual occupational tasks (Jakobsen et al., 2023). Similarly, exploratory field testing across several manufacturing environments identified perceived usability and user acceptance as relevant factors in the practical implementation of passive exoskeletons (Schwerha et al., 2022). More recent laboratory and field evaluations have also considered perceived effort, comfort, and acceptance when comparing passive back-support systems (Al-Khiami and Lindhard, 2026a). Therefore, future assessments should consider not only biomechanical outcomes but also comfort, perceived exertion, mobility, task compatibility, adaptation, and sustained willingness to use the device.

The distinction between laboratory and field conditions is particularly important when interpreting these results, as laboratory studies offer controlled conditions for quantifying biomechanical responses; however, field research has shown that prolonged occupational use can alter biomechanics and the user experience over time. Evidence of such biomechanical changes emerged from a study involving logistics workers who underwent field training with a passive back-support exoskeleton; a 24-week randomized controlled trial assessed long-term changes in biomechanics, work intensity, and musculoskeletal discomfort during actual occupational use (Jakobsen et al., 2025). These findings underscore the need for longitudinal field evaluations, as brief laboratory exposures may fail to capture adaptation processes, discomfort, changes in work technique, or other consequences that arise during repeated occupational use.

Overall, the evidence suggests that successful industrial implementation should be evaluated through a multidimensional framework that combines biomechanical effectiveness with unintended load redistribution, comfort, usability, worker acceptance, long-term adaptation, economic feasibility, and responsible management of worker-generated data. This broader perspective is necessary because a system may demonstrate substantial biomechanical assistance under controlled conditions while presenting practical limitations when used repeatedly during real occupational activities.

## 5 Conclusion

Sensor-integrated industrial exoskeleton systems represent a promising technological strategy for supporting ergonomic risk assessment by combining wearable sensing, biomechanical assessment, and intelligent data processing into integrated occupational monitoring frameworks. The available evidence indicates that these systems can enhance the objective evaluation of posture, muscle activity, fatigue, and biomechanical loading while supporting ergonomic decision-making across diverse industrial scenarios. However, their widespread and sustained occupational adoption has not yet been established, owing to the need for robust validation under real working conditions, standardized evaluation protocols, interoperable sensing architectures, and greater adaptation to worker and task variability. Future developments should prioritize human-centered, explainable, and scalable intelligent systems capable of providing continuous ergonomic monitoring and personalized assistance, thereby facilitating the responsible integration of sensor-enabled exoskeletons into Industry 5.0 environments and supporting sustained ergonomic risk reduction.

Nevertheless, the available evidence remains heterogeneous in terms of exoskeleton configuration, sensing architecture, participant characteristics, experimental protocols, outcome measures, and validation procedures. Most investigations were conducted under controlled laboratory or simulated conditions, whereas evidence from sustained real-world industrial deployment remains comparatively limited. Therefore, the current evidence supports reductions in biomechanical and ergonomic risk factors but does not establish long-term reductions in WMSD incidence. Industrial implementation is further constrained by worker and task variability, anthropometric adaptation, sensor placement and calibration, comfort and usability, environmental conditions, system robustness, interoperability, data-processing requirements, and the limited standardization of evaluation protocols. Future research should prioritize longitudinal and field-based validation, standardized biomechanical and ergonomic outcomes, interoperable multimodal sensing architectures, and robust artificial intelligence models validated across workers, tasks, and industrial environments. Human-centered, explainable, scalable, and adaptable designs should also address usability, reliability, data governance, and continuous performance monitoring. These developments can strengthen the evidence base for the occupational application of sensor-integrated industrial exoskeletons and clarify their contribution to ergonomic risk reduction and the reduction of WMSD-related risk.

## Data Availability

The original contributions presented in the study are included in the article/Supplementary Material, further inquiries can be directed to the corresponding author.
