# Supplementary material for: Sensor-integrated industrial exoskeletons for ergonomic risk reduction: a systematic review of biomechanical outcomes and intelligent control systems
Source: Front Bioeng Biotechnol. 2026 Oct 22;14:1952925. doi: 10.3389/fbioe.2026.1952925 (PMC13736717; doi:10.3389/fbioe.2026.1952925)
Supplement: Supplementary file 1 [file Supplementaryfile1.zip › Table S1. PRISMA_2020_checklist_Frontiers.docx]

| **Section and Topic** | **Item #** | **Checklist item** | **Location where item is reported** |
| --- | --- | --- | --- |
| **TITLE** | | |  |
| Title | 1 | Identify the report as a systematic review. | Page 1: Line 0 Sensor-Integrated Industrial Exoskeletons for Ergonomic Risk Reduction: A Systematic Review of Biomechanical Outcomes and Intelligent Control Systems |
| **ABSTRACT** | | |  |
| Abstract | 2 | See the PRISMA 2020 for Abstracts checklist. | Page 1: Line 23, and 36 |
| **INTRODUCTION** | | |  |
| Rationale | 3 | Describe the rationale for the review in the context of existing knowledge. | Page 2: Line 82 – 101 |
| Objectives | 4 | Provide an explicit statement of the objective(s) or question(s) the review addresses. | Page 2: Line 102 – 107 |
| **METHODS** | | |  |
| Eligibility criteria | 5 | Specify the inclusion and exclusion criteria for the review and how studies were grouped for the syntheses. | Page 4: Line 131 – 145 |
| Information sources | 6 | Specify all databases, registers, websites, organisations, reference lists and other sources searched or consulted to identify studies. Specify the date when each source was last searched or consulted. | Page 4: Line 146 – 153 |
| Search strategy | 7 | Present the full search strategies for all databases, registers and websites, including any filters and limits used. | Page 5: 179 – 206 |
| Selection process | 8 | Specify the methods used to decide whether a study met the inclusion criteria of the review, including how many reviewers screened each record and each report retrieved, whether they worked independently, and if applicable, details of automation tools used in the process. | Page 5: Line 191 – 193 |
| Data collection process | 9 | Specify the methods used to collect data from reports, including how many reviewers collected data from each report, whether they worked independently, any processes for obtaining or confirming data from study investigators, and if applicable, details of automation tools used in the process. | Page 7: Line 240 – 251 |
| Data items | 10a | List and define all outcomes for which data were sought. Specify whether all results that were compatible with each outcome domain in each study were sought (e.g. for all measures, time points, analyses), and if not, the methods used to decide which results to collect. | Page 7: Line 240 – 251 |
|  | 10b | List and define all other variables for which data were sought (e.g. participant and intervention characteristics, funding sources). Describe any assumptions made about any missing or unclear information. | Page 7: Line 252 – 263 |
| Study risk of bias assessment | 11 | Specify the methods used to assess risk of bias in the included studies, including details of the tool(s) used, how many reviewers assessed each study and whether they worked independently, and if applicable, details of automation tools used in the process. | Page 7: Line 252 – 263 |
| Effect measures | 12 | Specify for each outcome the effect measure(s) (e.g. risk ratio, mean difference) used in the synthesis or presentation of results. | Page 7: Line 252 – 263 |
| Synthesis methods | 13a | Describe the processes used to decide which studies were eligible for each synthesis (e.g. tabulating the study intervention characteristics and comparing against the planned groups for each synthesis (item #5)). | Section 3.6: Line 218 – 239 Figure 1 |
|  | 13b | Describe any methods required to prepare the data for presentation or synthesis, such as handling of missing summary statistics, or data conversions. | Page 7: Line 242 – 246  The evaluation focused on several key criteria, including the clarity of the research objectives, the transparency and reproducibility of the methodological procedures, the adequacy of the description of the industrial exoskeleton and its associated sensing technologies, the robustness of the biomechanical evaluation and validation methods, and the internal consistency of the reported findings. |
|  | 13c | Describe any methods used to tabulate or visually display results of individual studies and syntheses. | Page 7: Line 246 – 248  Each study was assessed using a structured scoring framework to ensure a systematic and comparable evaluation across publications. |
|  | 13d | Describe any methods used to synthesize results and provide a rationale for the choice(s). If meta-analysis was performed, describe the model(s), method(s) to identify the presence and extent of statistical heterogeneity, and software package(s) used. | Page 7: Line 248 – 251 Only studies that satisfied the predefined methodological quality threshold were retained for the final synthesis, ensuring that the review incorporated robust empirical evidence regarding the applications, biomechanical effectiveness, implementation challenges, and technological development of industrial exoskeletons for the prevention of work-related musculoskeletal disorders. |
|  | 13e | Describe any methods used to explore possible causes of heterogeneity among study results (e.g. subgroup analysis, meta-regression). | Page 7: Line 253 – 254  A risk of bias assessment was conducted to identify potential methodological limitations that could influence the reliability and interpretation of the findings reported in the selected studies. |
|  | 13f | Describe any sensitivity analyses conducted to assess robustness of the synthesized results. | Page 7: Line 254 – 258  The evaluation considered aspects related to study design, the transparency of experimental and technological validation procedures, the reliability of data collection methods, and the clarity with which exoskeleton configurations, associated sensing technologies, biomechanical evaluation protocols, and study outcomes were reported. |
| Reporting bias assessment | 14 | Describe any methods used to assess risk of bias due to missing results in a synthesis (arising from reporting biases). | Page 7: Line 258 – 260  Particular attention was given to the reproducibility of the methodological approaches and to the extent to which industrial exoskeletons were validated under real or simulated occupational environments. |
| Certainty assessment | 15 | Describe any methods used to assess certainty (or confidence) in the body of evidence for an outcome. | Page 7: Line 260 – 263  Studies presenting incomplete methodological descriptions, limited validation of exoskeleton performance, insufficient reporting of integrated sensing technologies, or inadequate biomechanical evaluation procedures were considered to present a higher potential risk of bias. |
| **RESULTS** | | |  |
| Study selection | 16a | Describe the results of the search and selection process, from the number of records identified in the search to the number of studies included in the review, ideally using a flow diagram. | Page 7 and Page 8: Table 1: Line 265 – 279  The included studies showed considerable heterogeneity in terms of occupational domains, targeted anatomical regions, mechanical actuation architectures, sensing technologies, and deployment settings. Most evaluations were conducted in simulated laboratory environments (TRL 4–5), with a strong representation of industrial, logistics, and manufacturing contexts. The evidence covered a wide range of sectors, including logistics, warehousing, manufacturing, automotive assembly, construction, general ergonomics assessment, healthcare, and agro-industry. |
|  | 16b | Cite studies that might appear to meet the inclusion criteria, but which were excluded, and explain why they were excluded. | Page 6: Line 219 – 226  The study selection process followed the sequential stages recommended by the PRISMA 2020 guidelines. The search and consultation of databases were completed on June 30, 2026, and the PRISMA flow diagram was generated using the online visualization tool available at https://estech.shinyapps.io/prisma_flowdiagram/. After duplicate removal, the remaining records entered the screening phase, in which two independent reviewers evaluated the titles and abstracts of the retrieved publications. This stage aimed to determine the relevance of each study with respect to the research objective, specifically focusing on publications addressing the development, implementation, or evaluation of biomechanical monitoring systems and intelligent architectures applied to industrial ergonomic assessment. As a result, 106 studies satisfied the methodological eligibility requirements and progressed to the final quality assessment. |
| Study characteristics | 17 | Cite each included study and present its characteristics. | Page 8: Table 1 Line 285 – 286 |
| Risk of bias in studies | 18 | Present assessments of risk of bias for each included study. | Page 7: Line 258 – 263  Particular attention was given to the reproducibility of the methodological approaches and to the extent to which industrial exoskeletons were validated under real or simulated occupational environments. Studies presenting incomplete methodological descriptions, limited validation of exoskeleton performance, insufficient reporting of integrated sensing technologies, or inadequate biomechanical evaluation procedures were considered to present a higher potential risk of bias. |
| Results of individual studies | 19 | For all outcomes, present, for each study: (a) summary statistics for each group (where appropriate) and (b) an effect estimate and its precision (e.g. confidence/credible interval), ideally using structured tables or plots. | Page 7: Line 266 – 271  The included studies showed considerable heterogeneity in terms of occupational domains, targeted anatomical regions, mechanical actuation architectures, sensing technologies, and deployment settings. Most evaluations were conducted in simulated laboratory environments (TRL 4–5), with a strong representation of industrial, logistics, and manufacturing contexts. The evidence covered a wide range of sectors, including logistics, warehousing, manufacturing, automotive assembly, construction, general ergonomics assessment, healthcare, and agro-industry. |
| Results of syntheses | 20a | For each synthesis, briefly summarise the characteristics and risk of bias among contributing studies. | Page 7: Section 4. 1: Line 272 - 279 The characteristics of the included studies are summarized in Table 1, which presents the main characteristics of the scientific literature (n = 106), detailing the main occupational domain, target body regions, performance design, detection systems and the Technology Readiness Level (TRL). The studies are organized by occupational domain—led by logistics and materials handling (29.2%), manufacturing and automotive (23.6%), and construction (16.0%)—and are further grouped by mechanical actuation architecture (such as passive elastic/spring systems, active motors, soft robotics, and non-exoskeleton postural assessment systems) to improve clarity and facilitate comparison between technological solutions. |
|  | 20b | Present results of all statistical syntheses conducted. If meta-analysis was done, present for each the summary estimate and its precision (e.g. confidence/credible interval) and measures of statistical heterogeneity. If comparing groups, describe the direction of the effect. | Page 7: Section 4. 1: Line 272 - 279  The characteristics of the included studies are summarized in Table 1, which presents the main characteristics of the scientific literature (n = 106), detailing the main occupational domain, target body regions, performance design, detection systems and the Technology Readiness Level (TRL). The studies are organized by occupational domain—led by logistics and materials handling (29.2%), manufacturing and automotive (23.6%), and construction (16.0%)—and are further grouped by mechanical actuation architecture (such as passive elastic/spring systems, active motors, soft robotics, and non-exoskeleton postural assessment systems) to improve clarity and facilitate comparison between technological solutions. |
|  | 20c | Present results of all investigations of possible causes of heterogeneity among study results. | Page 7: Section 4. 1: Line 272 - 279  The characteristics of the included studies are summarized in Table 1, which presents the main characteristics of the scientific literature (n = 106), detailing the main occupational domain, target body regions, performance design, detection systems and the Technology Readiness Level (TRL). The studies are organized by occupational domain—led by logistics and materials handling (29.2%), manufacturing and automotive (23.6%), and construction (16.0%)—and are further grouped by mechanical actuation architecture (such as passive elastic/spring systems, active motors, soft robotics, and non-exoskeleton postural assessment systems) to improve clarity and facilitate comparison between technological solutions. |
|  | 20d | Present results of all sensitivity analyses conducted to assess the robustness of the synthesized results. | Page 8: Line 280 - 285  Most of the included empirical evaluations were controlled laboratory experiments under simulated tasks, with a smaller proportion of field or in-situ trials (25.8% in logistics, 32.0% in manufacturing, and 47.1% in construction), and only agricultural studies showing full field deployment (100%). This distribution reflects the emerging nature of research in this field and highlights the predominance of controlled experimental settings over real-world industrial evaluations. |
| Reporting biases | 21 | Present assessments of risk of bias due to missing results (arising from reporting biases) for each synthesis assessed. | Page 14: Line 389 - 391  The most frequently reported limitations included the need for validation under real working conditions, larger and more heterogeneous study populations, and long-term evaluations to verify system performance during complete work shifts. |
| Certainty of evidence | 22 | Present assessments of certainty (or confidence) in the body of evidence for each outcome assessed. | Section 3 – section 4: Table 1  The characteristics of the included studies are summarized in Table 1, which presents the main characteristics of the scientific literature (n = 106), detailing the main occupational domain, target body regions, performance design, detection systems and the Technology Readiness Level (TRL). The studies are organized by occupational domain—led by logistics and materials handling (29.2%), manufacturing and automotive (23.6%), and construction (16.0%)—and are further grouped by mechanical actuation architecture (such as passive elastic/spring systems, active motors, soft robotics, and non-exoskeleton postural assessment systems) to improve clarity and facilitate comparison between technological solutions. |
| **DISCUSSION** | | |  |
| Discussion | 23a | Provide a general interpretation of the results in the context of other evidence. | Section 4.  Most of the included empirical evaluations were controlled laboratory experiments under simulated tasks, with a smaller proportion of field or in-situ trials (25.8% in logistics, 32.0% in manufacturing, and 47.1% in construction), and only agricultural studies showing full field deployment (100%). This distribution reflects the emerging nature of research in this field and highlights the predominance of controlled experimental settings over real-world industrial evaluations. |
|  | 23b | Discuss any limitations of the evidence included in the review. | Section 4:  The reviewed evidence demonstrates that sensor-integrated exoskeleton systems can reduce biomechanical exposure during physically demanding industrial activities, with reported decreases in muscle activation ranging from 13% to 68.2% and reductions in spinal loading between 8% and 34% depending on the assisted region and occupational task. |
|  | 23c | Discuss any limitations of the review processes used. | Section 4: Line 551 - 575  The current evidence indicates that the main research gap is the limited confirmation of long-term effectiveness under real industrial conditions. |
|  | 23d | Discuss implications of the results for practice, policy, and future research. | Section 4: Line 525 – 549  Future intelligent industrial exoskeletons should evolve from monitoring-oriented platforms toward adaptive cyber-physical systems capable of integrating real-time sensing, predictive models, and personalized assistance strategies. |
| **OTHER INFORMATION** | | |  |
| Registration and protocol | 24a | Provide registration information for the review, including register name and registration number, or state that the review was not registered. | Section 2.1. Lines 109-130:  Not applicable  This study adopted a structured literature review approach to examine the current state of scientific evidence on industrial exoskeletons for preventing work-related musculoskeletal disorders across industrial environments. To ensure transparency, reproducibility, and a systematic identification and selection of the available evidence, the review process followed the methodological recommendations of the PRISMA 2020 statement. |
|  | 24b | Indicate where the review protocol can be accessed, or state that a protocol was not prepared. | Section 2.6: Line 239 – 251  The selection procedure adhered strictly to the four phase PRISMA 2020 protocol: identification, screening, eligibility, and final inclusion <https://estech.shinyapps.io/prisma_flowdiagram/> |
|  | 24c | Describe and explain any amendments to information provided at registration or in the protocol. | No amendments to the registered protocol were mad |
| Support | 25 | Describe sources of financial or non-financial support for the review, and the role of the funders or sponsors in the review. | This research was funded by the Universidad Nacional del Santa under the Call for Competitive Grants for Research Projects Financed with Mining Royalty Resources, Research Groups Category - 2023, pursuant to Resolution No. 749-2023-CU-R-UNS and Contract No. 0022-2025-UNS-OAJ, corresponding to the project “Development of an intelligent biomechanical system in fishing sector workstations to improve job performance’’ |
| Competing interests | 26 | Declare any competing interests of review authors. | “The authors declare no conflicts of interest.” |
| Availability of data, code and other materials | 27 | Report which of the following are publicly available and where they can be found: template data collection forms; data extracted from included studies; data used for all analyses; analytic code; any other materials used in the review. | Statement on data availability: “All data generated or analyzed during the study took into account the selected quality procedure, where characteristics of high-impact databases were considered, including the document identifier and its digital registration in the Scopus and Web of Science databases.” |

*From:*  Page MJ, McKenzie JE, Bossuyt PM, Boutron I, Hoffmann TC, Mulrow CD, et al. The PRISMA 2020 statement: an updated guideline for reporting systematic reviews. BMJ 2021;372:n71. doi: 10.1136/bmj.n71. This work is licensed under CC BY 4.0. To view a copy of this license, visit <https://creativecommons.org/licenses/by/4.0/>
