# Supplementary material for: Sensor-integrated industrial exoskeletons for ergonomic risk reduction: a systematic review of biomechanical outcomes and intelligent control systems
Source: Front Bioeng Biotechnol. 2026 Oct 22;14:1952925. doi: 10.3389/fbioe.2026.1952925 (PMC13736717; doi:10.3389/fbioe.2026.1952925)
Supplement: Supplementary file 1 [file Supplementaryfile1.zip › TABLE S2.docx]

**TABLE S2.** PECO framework aligned with the research objective

| **PECO Component** | **Analytical Scope** | **Keywords Included in Search** |
| --- | --- | --- |
| Population | Industrial workers performing physically demanding tasks in manufacturing, construction, logistics, healthcare, and other occupational environments where industrial exoskeletons are applied to prevent work-related musculoskeletal disorders. | Industrial ergonomics, occupational ergonomics, industrial workplace, work-related musculoskeletal disorders, WMSDs, occupational safety. |
| Exposure | Industrial exoskeletons (passive, active, soft, and powered) together with the intelligent sensing, biomechanical assessment, and computational technologies used to evaluate and optimize their ergonomic performance, including electromyography, inertial measurement units, motion capture, computer vision, biomechanical modeling, digital human modeling, artificial intelligence, and sensor fusion. | Industrial exoskeleton, occupational exoskeleton, wearable exoskeleton, exoskeleton, EMG, sEMG, IMU, motion capture, computer vision, machine learning, artificial intelligence, biomechanical modeling, digital human model, sensor fusion. |
| Comparator | Conventional work without exoskeleton assistance, traditional ergonomic assessment methods, or comparisons among different exoskeleton designs, assistance mechanisms, and biomechanical evaluation approaches. | Ergonomic risk assessment, musculoskeletal risk assessment, RULA, REBA, OCRA, NIOSH lifting equation. |
| Outcome | Biomechanical effectiveness, reduction of muscle activity and spinal loading, ergonomic performance, occupational applications, implementation challenges, user acceptance, technological limitations, and future research trends associated with industrial exoskeletons. | Ergonomic assessment, biomechanical performance, muscle activity, fatigue detection, posture assessment, implementation challenges, validation, occupational applications, industrial deployment, future trends. |
